# Application of a Multi-Satellite Dynamic Mission Scheduling Model Based on Mission Priority in Emergency Response

**DOI:** 10.3390/s19061430

**Published:** 2019-03-23

**Authors:** Jintian Cui, Xin Zhang

**Affiliations:** 1 State Key Laboratory of Remote Sensing Science, Aerospace Information Research Institute, Chinese Academy of Sciences, Beijing 100101, China; cuijt@radi.ac.cn; 2 University of Chinese Academy of Sciences, Beijing 100101, China

**Keywords:** mission priority, Earth observation satellite, satellite mission scheduling, emergency response, multi-constraint optimization problem, hybrid genetic tabu search algorithm

## Abstract

Emergency observations are missions executed by Earth observation satellites to support urgent ground operations. Emergency observations become more important for meeting the requirements of highly dynamic and highly time-sensitive observation missions, such as disaster monitoring and early warning. Considering the complex scheduling problem of Earth observation satellites under emergency conditions, a multi-satellite dynamic mission scheduling model based on mission priority is proposed in this paper. A calculation model of mission priority is designed for emergency missions based on seven impact factors. In the satellite mission scheduling, the resource constraints of scheduling are analyzed in detail, and the optimization objective function is built to maximize the observation mission priority and mission revenues, and minimize the waiting time for missions that require urgency for execution time. Then, the hybrid genetic tabu search algorithm is used to obtain the initial satellite scheduling plan. In case of the dynamic arrival of new emergency missions before scheduling plan releases, a dynamic scheduling algorithm based on mission priority is proposed to solve the scheduling problem caused by newly arrived missions and to obtain the scheduling plan of newly arrived missions. A simulation experiment was conducted for different numbers of initial missions and newly arrived missions, and the scheduling results were evaluated with a model performance evaluation function. The results show that the execution probability of high-priority missions increased because the mission priority was taken into account in the model. In the case of more satellite resources, when new missions dynamically arrived, the satellite resources can be reasonably allocated to these missions based on the mission priority. Overall, this approach reduces the complexity of the dynamic adjustment and maintains the stability of the initial scheduling plan.

## 1. Introduction

The mission scheduling of Earth observation satellites is performed to determine the execution order, execution time and corresponding satellite resources in the case of a fixed number of satellites and limited storage capacity. In the process of scheduling, it is necessary to eliminate the timing conflicts between different missions, improve the utilization of satellite resources and maximize the needs of users. In recent years, with the wide application of Earth observation satellites in emergency conditions, such as natural disaster monitoring, accident disasters and public health incidents, the scheduling of the Earth observation satellite has faced some new challenges. There are many uncertainties in the scheduling of Earth observation satellites under emergency conditions, including the user needs, weather conditions, and the satellite status [1]. In addition, emergency missions submitted by users usually arrive dynamically, and the number and arrival time of missions are uncertain. Due to the characteristics of dynamic real-time scheduling, multiple objectives need to be taken into account simultaneously, such as scheduling revenue, stability and energy conservation. All of these constraints and uncertainties make emergency scheduling an NP-hard combinatorial optimization problem [2]. Therefore, it is of great significance to propose a fast and efficient multi-satellite dynamic emergency scheduling strategy.

Recently, some scholars have carried out extensive research on satellite scheduling problems. Under the condition of satisfying multi-resource constraints, the satellite resources and observation period are allotted for imaging observation missions in order to improve the efficiency of mission execution. However, in taking the resources contention into account, it is more complicated and challengeable to reasonably assign multiple satellites to targets [3]. The methods used to solve this problem include priority-based heuristics [4], local search [5], tabu search [6,7], genetic algorithms [2] and ant colony algorithms [8]. Muraoka et al. [9] used greedy algorithm for satellite imaging scheduling in the ASTER system. Bianchessi et al. [10] indicated that a greedy algorithm can provide approximately optimal results in a permissible time and is commonly used in solving relevant problems. However, the algorithm performance is sensitive to the heuristic strategies. The aforementioned satellite scheduling strategies are proposed in the framework of static scheduling, which is not applicable to multi-satellite dynamic scheduling problems under emergency conditions. For dynamic emergency scheduling, one remarkable feature is the arrival of new emergency imaging missions. Some scholars have used the complete reprogramming algorithm to remodel and solve this problem [11,12,13]. However, the new mission scheduling plan generated is greatly different from the original one, which leads to difficulty in satellite rescheduling. Perberton et al. [14] summarized four factors that can result in changes to the satellite mission scheduling plan: mission observation opportunity changes, resource changes, new mission insertion, and scheduling parameter changes. However, no specific model or algorithm was presented. Verfaillie et al. [15] proposed a satellite dynamic mission scheduling model based on the dynamic constraint satisfaction problem (DCSP), but did not consider the adjustability of the initial plan. Wang et al. [16] presented a novel task dynamic merging strategy, and retracting and rearranging operations were conducted to make room for incorporating the newly arrived missions. He et al. [17] proposed a hierarchical scheduling method based on an ant colony algorithm for the real-time scheduling problem, but did not consider observation requirements, image data type or acquisition. Considering the real-time satellite scheduling and arrival of new missions, Sun et al. [18] proposed a dynamic scheduling algorithm based on constraint satisfaction, but the efficiency of this method needs to be further improved.

The satellite scheduling for emergency missions is different from that for conventional observations. Emergency missions have more stringent time constraints and require observations in a limited visible time window before the cut-off time. Liu et al. [19] proposed a spatial optimization model to solve the large-area satellite image acquisition planning problem in the context of hazard emergency response, and the priority of satellite image acquisition for different portions of impact areas were addressed. Niu et al. [20] expressed the multi-satellite tasking problem as a multi-objective integer-programming model, including optimizing objectives of the coverage rate, the imaging completion time, the average spatial resolution and the average slewing angle. A real disaster scenario was revisited in terms of satellite image acquisition in the context of emergency response. However, these methods mainly focuses on the acquisition of satellite images in emergency situations, and there are no detailed methods regarding the problem of satellite scheduling. In addition, due to the differences in the importance of missions, the order of execution varies, so a reasonable and effective priority algorithm is important for improving the efficiency of mission execution. Most scholars study satellite networks by setting the mission priority to evaluate the revenues of scheduling and regard the priority as the main optimization objective. Sarkheyli et al. [21] applied a new tabu search algorithm and addressed a scheduling problem for LEO satellite missions, assigning satellites to the greatest number of requested missions by considering the mission priority and satisfying multi-resource constraints. In terms of solution algorithms, there is no universal algorithm for all problems since the satellite mission scheduling problem involves a large number of non-linear constraints and the solution objectives are not unique. Frank et al. [22] proposed a constraint-based model and a description method for the scheduling of Earth observation satellites. Globus et al. [23] studied the multi-satellite joint model and applied genetic algorithm to solve the problem. Mission priority was taken into account in this model, and each satellite has constraint condition, but factors such as the storage capacity limitation and data downlink were not taken into account. Malladi et al. [24] introduced the Clustered Maximum Weight Clique Problem (CCP) which was derived from the Satellite Image Acquisition Scheduling Problem (SIASP), and proposed a novel model-based matheuristic which took advantage of the power of the modern mixed-integer programming solvers and boosted the performance by including appropriate constraints. For optimization objective function, Tangpattanakul et al. [25] presented an indicator-based multi-objective local search method to solve a multi-objective optimization problem, which was to maximize the total revenue of the selected acquisitions and simultaneously ensure the fair resource sharing by minimizing the maximum revenue difference among users. Malladi et al. [26] studied the image acquisition scheduling for the Canadian RADARSAT Constellation Mission (RCM). An integer programming model was proposed to employ a piecewise linear objective function to favor completion of an image acquisition request and has achieved good results. Wang et al. [27] proposed a heuristic algorithm based on minimum resource competition with the objective of minimizing scheduling costs and maximizing mission revenues. However, most of the methods above were based on general missions and are difficult to apply in emergency mission scheduling.

Guided by the application requirements of emergency observation, this paper focuses on the key technologies of the multi-satellite dynamic scheduling problem and employs the proposed mathematic model and algorithm in an automatic mission control system for constellations of satellites. The overall objective of this study is to design a mission-oriented satellite scheduling architecture under emergency conditions based on the following goals: (1) to design priority evaluation criteria for emergency missions. The seven impact factors that influence the mission priority, including imaging mission level, type of observation images, visibility of target to satellite, execution urgency degree, type of mission, mission conflict degree and revenue of imaging mission, are taken into account to obtain the priority level of mission, which is then used as heuristic information for solving constraint scheduling problem; (2) to analyse the main constraints of satellite scheduling and the objective function in detail. Each term of the objective functions is maximizing the mission priority, maximizing the mission revenue and minimizing waiting time for missions that require an urgent execution time. Then, the hybrid genetic tabu search algorithm is used to obtain the initial satellite scheduling plan, so as to avoid useless iterations when close to the best solution in the late stage of algorithm implementation; and (3) to develop a dynamic scheduling algorithm based on mission priority to solve the scheduling problem in case of newly arrived missions. In this approach, newly arrived missions are added into the initial scheduling plan to obtain a new scheduling plan. The validity of the algorithm is verified through experimental simulations, and the feasibility of the emergency observation mission scheduling model is verified based on a model performance evaluation function.

## 2. Methodology

### 2.1. Multi-Satellite Dynamic Mission Scheduling Model Based on Mission Priority under Emergency Conditions

**Figure 1:**
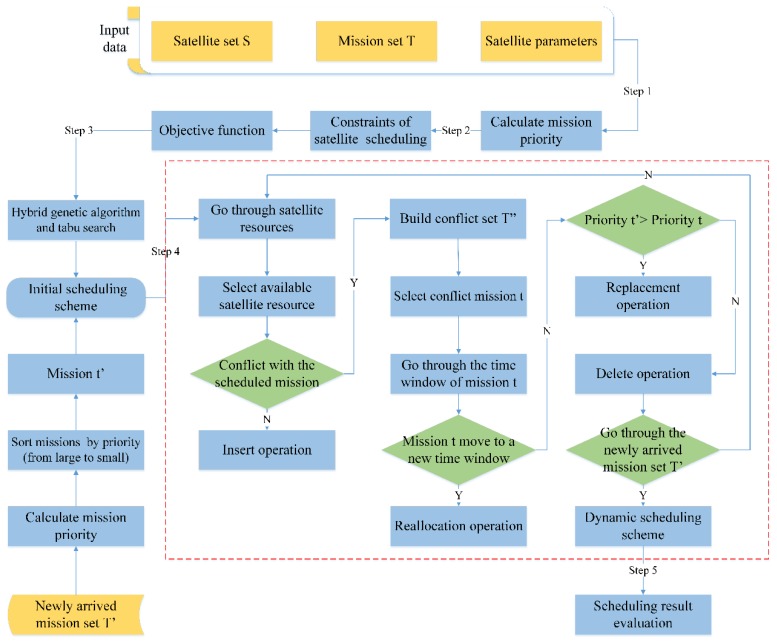
Flow chart of a multi-satellite dynamic mission scheduling model based on mission priority under emergency conditions.

In emergency observation, there are many factors that need to be considered to maximize a total reward of the observed targets, such as the priority level of missions and the total benefit gained from acquiring images. Furthermore, in contrast to daily scheduling, the resource allocation for real-time missions as an important feature of this problem also need to be addressed. In the context of a large number of point target imaging missions with different priority levels and newly arrived missions, we develop a multi-satellite dynamic mission scheduling model based on the mission priority under emergency conditions. The algorithm includes the following steps: (1) analysing the factors that influence the mission priority and calculating the priority for emergency missions; (2) analysing the constraints of satellite scheduling and building an optimization objective function; (3) obtaining the initial scheduling plan with the hybrid genetic tabu search algorithm; (4) using dynamic scheduling algorithm tries to insert newly arrived missions to the initial scheduling plan; and (5) evaluating the scheduling plan results. The flow chart of this model is shown in Figure 1.

### 2.2. Sequencing the Mission Priority 

#### 2.2.1. Impact Factors of Mission Priority

Due to the limitation of satellite resources and different users’ needs for remote sensing data, the priority level of emergency missions varies. By analyzing the application requirements of emergency missions, the following seven impact factors are considered in setting the mission priority (Table 1).

(1) Imaging mission level. In the process of emergency response, different emergencies can be divided into four categories: natural disaster, accident disaster, public health incident and social security incident. According to the nature, severity, controllability and range of influence, it can be divided into four levels (I–IV), and the emergency response mechanism decreases from level I to level IV. Therefore, the corresponding imaging mission level is divided into four levels, and the missions with high level of this factor are given high priority.
(2) Type of observation image. Satellite scheduling for emergency missions involves multiple types of remote sensors, thus different images can be selected by users according to their needs. Therefore, the types of remote sensing images can be employed for calculating the priority level of mission. We take visible light, infrared and microwave images as examples in this study, and assume that the image types are ordered as follows: visible light image > microwave image > infrared image.
(3) Visibility of target to satellite. During satellite scheduling, there are cases in which the same target is within multiple satellite observation ranges at the same time. For a mission, the visibility of target to satellite is related to the number of available satellites in the same time window, and the visibility between the satellite and target can be computed by calling satellite tool kit (STK) software. Therefore, a mission with less available satellite resources should be given high priority, thus to ensure the execution of the mission. Besides, when no satellite can serve the mission, the mission is declared invalid and removed from the list of missions that need to be scheduled.
(4) Execution urgency degree. Emergency observations have different response time requirements. The start time, end time and remaining time of a mission determine whether it could be successfully carried out and completed. Therefore, the more urgent the mission is, the more likely it is to miss the execution period and fail, so it should be given priority for execution.
(5) Type of mission. Due to the emergency missions with different characteristics, the types of missions should be classified and given different priorities. Especially, the position of maritime target has great uncertainty and always changes with time, so it needs to be observed first. Therefore, the importance of different mission types is defined as the following sequence: maritime moving target > maritime static target > land moving target > land static target.
(6) Mission conflict degree. In some cases, there may be multiple missions requesting a certain satellite resource at the same time, resulting in resource contention. Therefore, we define the conflict degree of a mission as the number of missions competing for the same satellite resource. The mission with a high conflict degree should be given priority for execution in order to avoid the problem of deadlock during scheduling.
(7) Revenue of imaging mission. A basic objective of mission scheduling is to maximize the total revenue gained from acquiring images. For imaging satellites, meteorological conditions, especially cloud cover, have a great influence on imaging effect. Therefore, the total mission revenue depends not only on the value of the mission, but also on the imaging quality. Therefore, we adopt the basic revenue which is related to the importance of the observation missions and cloud cover which is related to the imaging quality to measure the total benefit.

#### 2.2.2. Calculation Model of Mission Priority

For the impact factors of mission priority, the above qualitative impact factors should be quantified in the calculation model. We used technique for order preference by similarity to ideal solution (TOPSIS) [29,30,31] to perform a synthetic calculation and obtain quantitative indicators of mission priority. The implementation of our method is as follows:

Step 1: For mission set $T=\{t_{1},t_{2},\ldots ,t_{m}\}$, the impact factors of mission priority are calculated to form an impact factor matrix $X={\left[x_{ij}\right]}_{m\times 7}$, where $x_{ij}$ is the quantitative value of the j-th impact factor of mission $t_{i}$, and *m* is the number of missions.

Step 2: The range transformation method is used to transform the impact factor matrix into the standard matrix $Y={\left[y_{ij}\right]}_{m\times 7}$, $0\leq y_{ij}\leq 1$:

(1) $$y_{ij}=\frac{x_{ij}-x_{\min}}{x_{\max-}x_{\min}}$$

where $x_{\max}$ is the maximum value in the column that contains $x_{ij}$ and $x_{\min}$ is the minimum value in the column that contains $x_{ij}$.

Step 3: The ideal solution is set as $I=<I^{+},I^{-}>$, where $I^{+}=(1,1,\ldots ,1)$ is the positive ideal solution and $I^{-}=(0,0,\ldots ,0)$ is the negative ideal solution.

Step 4: Calculate the close-degree $C_{i}$ between mission $t_{i}$ and the ideal solution:

(2) $$\begin{array}{c} C_{i}=\frac{D_{i}^{-}}{D_{i}^{-}+D_{i}^{+}}\\ D_{i}^{+}=\sqrt{\sum_{j=1}^{n}{\left(y_{ij}-1\right)}^{2}}\\ D_{i}^{-}=\sqrt{\sum_{j=1}^{n}{\left(y_{ij}-0\right)}^{2}} \end{array}$$

where $D_{i}^{+}$ and $D_{i}^{-}$ are the positive and negative distances between mission $t_{i}$ and the ideal solution I, and *n* is the number of impact factors.

Step 5: The priority level of mission *t_i_* is measured by *p_i_*:

(3) $$p_{i}=\left\lfloor C_{i}\right\rfloor \times 10$$

The priority level of each mission can be obtained through the above steps, and the level is in the range of [0, 10]. The higher the value of *p_i_* is, the higher the priority of the mission.

### 2.3. Emergency Observation Mission Scheduling Model

In contrast to the traditional scheduling model, dynamic emergency scheduling is mainly for non-periodic missions with uncertain expected completion and arrival time. Under the conditions of satisfying the satellite observation constraints and mission requirements, this paper builds a multi-objective mathematical programming model for multi-satellite mission scheduling under emergency conditions, so as to maximize the objective of mission scheduling and optimize the observation plan.

#### 2.3.1. Constraint Analysis of Satellite Scheduling

The mission defined in this paper refers to the point target that can be observed by the satellites in a field of view. Considering multi-resource constraints, we established a mathematical model to describe the scheduling problem. In the observation scheduling stage, the main constraints are as follows. The parameter and label definitions in the constraints are listed in Table 2, and function definitions are shown in Table 3.

(1) Visible window constraint. Satellite payloads and observation targets must be visible:
  (4) $$\begin{array}{c} \forall tm\in ST_{ij},\exists l:p_{ijl}(tm),\\ bst_{ij}\geq bos_{ij}^{l},est_{ij}\leq eos_{ij}^{l},\\ est_{ij}-bst_{ij}\geq CapLen(t_{i}) \end{array}$$
(2) Start-up time constraint. The start-up time must be between the minimum start-up time *MinT_j_* and the maximum start-up time *MaxT_j_* of sensor $s_{j}^{\prime }$ of satellite *s_j_*. The time interval between two switches cannot exceed the preset minimum time interval *MinPeriod_j_*.
  (5) $$\begin{array}{c} \forall i=[1,m],\forall j=[1,n],\\ CS(OS_{ij})-IS(OS_{ij})\geq MinT_{j},\\ CS(OS_{ij})-IS(OS_{ij})\leq MaxT_{j},\\ IS(OS_{ij})-CS(OS_{(i-1)j})\geq MinPeriod_{j} \end{array}$$
(3) Solar elevation angle constraint. For a visible light payload, sensor $s_{j}^{\prime }$ of satellite *s_j_* must satisfy the constraint of the solar elevation angle *Sunθ_i_*:
  (6) $$\forall i,j,tm:SunAngle(t_{i},tm)\geq Sun\theta_{i},tm\in ST_{ij}$$
(4) Observation angle constraint. The satellite payload and observation mission must meet the minimum observation angle requirement *MinObserθ_i_* specified by the user:
  (7) $$\forall i,j,tm:ObserAngle(t_{i},s_{j},tm)\geq MinOber\theta_{i},tm\in ST_{ij}$$
(5) Storage capacity constraint. For satellite *s_j_*, the storage capacity of the observation mission between any two adjacent data transmission windows *w*_1_, *w*_2_ cannot exceed the storage capacity *M_j_* of satellite *s_j_*:
  (8) $$\begin{array}{c} \forall j\in [1,n],\forall w_{1},w_{2}\in GSW_{j},w_{1}\neq w_{2},\\ \sum \left(StorCap(t_{1},v_{j})+StorCap(t_{2},v_{j})+\ldots +StorCap(t_{i},v_{j})\right)<M_{j} \end{array}$$
(6) Energy constraint. The energy consumed by satellite activities can be expressed as a correlation function of observation time, sensor side swing and on and off processes. The consumption cannot exceed the maximum limit *P_j_*. $P_{j}^{\prime }$ is defined as the energy consumed per unit time of observation, *Q_j_* is the energy consumed per unit angle of side swing, and *O_j_* is the energy consumption associated with turning on and off the machine:
  (9) $$\sum_{i=1}^{m}(eos_{ij}^{l}-bos_{ij}^{l})\cdot P_{j}^{\prime }+\sum_{i=1}^{m}\left|SA_{(i+1)j}^{l}-SA_{ij}^{l}\right|\cdot Q_{j}+NUM(E)O_{j}\leq P_{j}$$
(7) Satellite payload action constraint. For sensor $s_{j}^{\prime }$ of satellite *s_j_*, the time interval of continuous observation missions cannot be less than the time of side swing adjustment and the time of stability after a side swing. *V_j_* is the speed of the side swing, which is simplified to a constant speed, and *ts_j_* is the time of stability after a side swing:
  (10) $$\begin{array}{c} \forall i_{1},i_{2}\in [1,m],i_{1}\neq i_{2},\forall j\in [1,n],\forall l_{1},l_{2}\in N_{ij},\\ bos_{i_{2j}}^{l_{2}}-eos_{i_{1}j}^{l_{1}}>|SA_{i_{1}j}^{l_{1}}-SA_{i_{2j}}^{l_{2}}|/V_{j}+ts_{j} \end{array}$$
(8) Imaging mode constraint for radar satellites. For a radar satellite payload, the working wave cannot be changed during the imaging process.
  (11) $$\begin{array}{c} \forall t_{i}\in T,mt_{ia},mt_{ib}\in MT_{i},a\neq b,\\ WP(mt_{ia})=WP(mt_{ib}) \end{array}$$

#### 2.3.2. Optimization Objective Function of Satellite Scheduling

The satellite mission scheduling is in reality a multi-objective optimization problem with multiple constraints. During emergency response, it not only requires that the completion rate of key emergency missions is high and the mission revenue is large, but also needs to ensure a high scheduling efficiency for each mission to optimally use satellite resources. Based on these consideration, we use a linear weighted sum method to build optimization objective function for solving this model:

(1) Sub-objective function 1: to maximize the sum of priority levels of the missions:
  (12) $$Maximize\;f_{1}=\sum_{j=1}^{n}\sum_{i=1}^{m}\sum_{k=1}^{k_{ij}}x_{ijk}p_{i}$$
  where $X={\left\{x_{ijk}\right\}}_{m\times n\times k_{ij}}$ is the decision matrix of mission scheduling, *x_ijk_* = 1 represents the *k*-th observation opportunity of mission *t_i_* assigned to satellite *s_j_*; otherwise, *x_ijk_* = 0.
(2) Sub-objective function 2: to maximize the total mission revenues:
  (13) $$Maximize\;f_{2}=\sum_{j=1}^{n}\sum_{i=1}^{m}\sum_{k=1}^{k_{ij}}x_{ijk}w(t_{i})$$
(3) Sub-objective function 3: to minimize the waiting time for missions that require urgent execution:
  (14) $$Minimize\;f_{3}=\sum_{j=1}^{n}\sum_{i=1}^{m}\sum_{k=1}^{k_{ij}}(bst_{i}-tsb_{i})/(tse_{i}-tsb_{i})\cdot urgent(t_{i})\cdot x_{ijk}$$

Based on each term of the objective functions, the global optimization objective function is obtained by the linear weighted sum method:

(15) $$Maximize\;f=\alpha \cdot f_{1}+\beta \cdot f_{2}+\gamma \cdot 1/f_{3}$$

where *α, β* and *γ* are the weighting factors, and the sum of these factors is 1. The value of the weighting factor determines the importance of each term in the objective function. Our study takes the mission priority as the key factor in the emergency response. Thus, we set *α* *=* 0.6, *β* = 0.2 and *γ* = 0.2. 

### 2.4. Dynamic Allocation of Satellite Resources for Emergency Scheduling

The purpose of emergency observation mission scheduling is to determine the execution order of the missions and reasonably allocate satellite resources and execution time for each mission. As some new emergency missions are added, the complexity of the problem is further increased. Because there are many constraints in the multi-satellite scheduling problem, and the arrival of new missions will affect the mission set, satellite resource set, time window and constraints in the initial model, thus influencing many scheduled missions. Therefore, this paper proposes a dynamic scheduling algorithm based on mission priority to solve the scheduling problem of newly arrived missions. In this model, the initial schedule is obtained through a hybrid genetic tabu search algorithm. When new missions arrive, based on the initial scheduling results, the satellite that matches the new mission request is searched in the satellite resource set, the operations including insertion, reallocation, replacement and deletion are performed to execute the new missions with high priority. We design this method to ensure that newly arrived missions can be quickly scheduled under emergency conditions and minimize the change of initial scheduling plan.

#### 2.4.1. Initial Scheduling Plan of Satellite Resources

The genetic algorithm and tabu search are common algorithms for solving combinatorial optimization problems [32,33,34]. The genetic algorithm is a highly parallel, stochastic and adaptive optimization algorithm based on the biological evolution and selection mechanism, and it has strong robustness and high global search capability. However, in the late stage, the convergence speed and the computational efficiency are low, and it is easy to converge to a local optimum. Tabu search is a neighborhood search algorithm that prohibits the repetition of previous work and avoids the local optimum. However, tabu search also has some limitations, such as strong dependence on the initial solution, serial operation for only one solution, and low search efficiency. 

Based on the characteristics above, a hybrid genetic tabu search algorithm is designed in this paper to allocate satellite resources to the mission set. The algorithm is used to solve the combinatorial optimization model and obtain the initial scheduling plan. The core concept of this algorithm is as follows: (1) the genetic algorithm is used for large-scale searches in the global space. The initial population traverses most of the solution space in parallel. At the end of iteration, the result is stable in the region where the solution space is better; and (2) starting from each individual in the optimization region, the tabu search is applied in a small-scale search of the local space to delay or avoid the genetic algorithm converging to a local optimum, thereby improving the ability to search for the best solution. The process of the hybrid genetic tabu search algorithm is shown in the following processing chain (Table 4):

#### 2.4.2. Dynamic Scheduling of Newly Arrived Emergency Missions 

This section presents the dynamic scheduling algorithm which tries to insert newly arrived missions into the initial scheduling plan. The newly arrived missions are sorted by priority, from high to low, and scheduled one by one. The scheduling operations are applied to each new mission until one of the operations is successful. The operations are: insertion, reallocation, replacement, and deletion. The algorithm stops when all new missions are attempted to be scheduled, and an ideal dynamic mission scheduling plan is obtained by iteration according to the proposed method. The details of the scheduling operations are as follows:

Step 1: Initialize the parameters. The missions in the new mission set *T’* are sorted from high to low according to mission priority, so that the mission with the highest priority in *T’* is the current scheduling mission *t’* with available satellite resource set *S*.
Step 2: Perform the insertion operation. This step involves traversing every available satellite resource of *S* for mission *t’* to determine whether the visible time window of the current satellite resource *S’* is available and whether there is a conflict between missions *t’* and the scheduled mission in the current time window of satellite *S’*. If there is no conflict, the mission *t’* is directly inserted into the current time window. If the new mission cannot be inserted, mission *t* which conflicts with mission t’ is placed in the mission conflict set *T*”, and the process proceeds to step 3.
Step 3: Perform the reallocation operation. This step traverses all missions in the conflict set *T”* that conflict with the current mission *t’*, makes the first mission in the conflict set as the current conflict mission *t*, and re-searches its available time window. If the current conflicting mission *t* can be moved to a new window, mission *t‘* is inserted into the current time window, and the conflicting mission *t* is moved to another time window for execution. If the current conflicting mission *t* cannot be moved to a new window, then the process proceeds to step 4.
Step 4: Perform the replacement operation. This step assesses the priority of mission *t’* and the current conflicting mission *t*. If the priority of mission *t‘* is higher than that of mission *t*, the current conflicting mission *t* is deleted, and then the new mission *t’* is inserted into the visible time window; otherwise, the mission replacement is unsuccessful, and the process proceeds to step 5.
Step 5: Perform the delete operation. When steps 2, 3 and 4 are not satisfied, the new mission *t’* is deleted and the resource scheduling process is abandoned.
Step 6: Determine whether the available satellite resources are traversed. If the available resources have been traversed, the solution corresponding to the maximum value in the evaluation set is selected as the final solution, and the scheduling of current mission *t’* is completed. At this point, the process proceeds to step 1 until all new missions in the set *T’* are scheduled; otherwise, the satellite resource number plus 1 and the process proceeds to step 2.

### 2.5. Model Performance Evaluation

The multi-satellite dynamic emergency scheduling model based on mission priority not only requires a high mission completion rate and high total revenues, but also needs to ensure a better scheduling period for each mission and a high execution rate of missions with high priority. According to the characteristics of the model, three indicators are defined to evaluate the model performance, including the mission completion rate (*MCR*), mission priority execution rate (*MPER*), and scheme change rate (*SCR*). The evaluation function of the model is given in Equation (16):

(16) $$\begin{array}{c} f(u)=\frac{MCR\times MPER}{SCR}\\ MCR=n/n^{\prime }\\ MPER=\sum_{i=1}^{n}p_{i}/\sum_{i=1}^{n^{\prime }}p_{i}\\ SCR=num(T_{c})/num(T_{q}) \end{array}$$

where *n* is the total number of actual scheduled missions after scheduling plan adjustment, *n’* is the total number of missions, *num*(*T_c_*) is the number of missions that the initial scheduling plan changes due to the newly arrived missions in dynamic scheduling, and *num*(*T_q_*) is the number of initial missions.

## 3. Results

According to the proposed method, we used Microsoft Visual Studio 2010 as the development environment, and coded the algorithm in C#. The experiments were performed using an Inter (R) Core (TM) i5-3317U 1.70 GHz CPU with 4 GB RAM under the Windows 7 operating system. The setup and results of the experiments will be discussed in this section.

### 3.1. Simulation Experiment Setting

**Figure 2:**
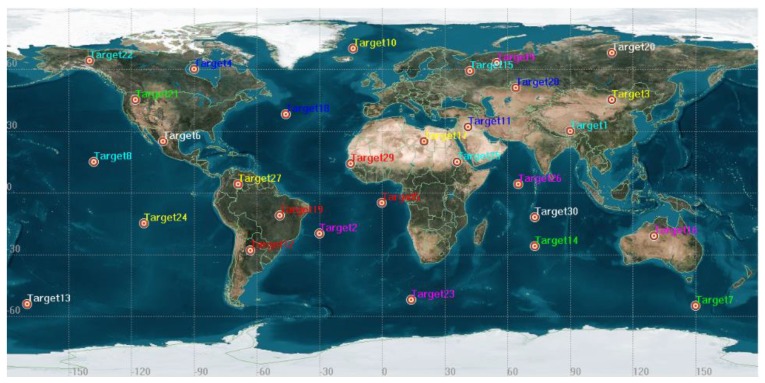
Point target distribution.

In the test scenario, mission request is generated by a random uniform distribution in worldwide. For each request of emergency mission, the following attributes are included: observation duration, geographical location, imaging mission level, observation image type and type of mission. In this paper, 25 initial observation point targets were randomly generated, as shown in Figure 2, and the generated mission types are shown in Table 5. Using the same method, we generated another five as the newly arrived missions to test the dynamic scheduling algorithm (Table 6). 

The time horizon of scheduling was from 2018/08/24 00:00 to 2018/08/24 14:00 (universal time coordinated), and the imaging duration was varied from 60 to 240 s. For satellite resources, three Earth observation satellites were set up in this experiment. These satellites were taken from the satellite database of STK software developed by AGI of the United States. The orbit parameters are shown in Table 7 and were generated calling the orbital generation tool of STK according to the general orbit type of Earth observation satellite.

### 3.2. The Calculation of Mission Priority

According to the seven types of mission priority impact factors discussed in Section 2.2.1, the relevant information for the five newly arrived missions after pretreatment is shown in Table 8. The observation time window of the satellite for each point target was calculated by calling STK software. Firstly, the qualitative descriptions in the impact factors table were transformed into quantitative descriptions. The visible light, microwave and infrared image types were assigned values of 1, 2, and 3, respectively, and the maritime moving target, maritime static target, land moving target and land static target were assigned values of 1, 2, 3, and 4, respectively. For the impact factors, F1, F3, F6 and F7 are positive indicators, and F2, F4 and F5 are negative indicators. Therefore, the reciprocal method was used to convert all negative indicators into positive indicators. Then, the range transformation method was used to convert the transformed impact factor matrix into the standard matrix (Table 9). The distance between each mission and the ideal positive solution and the ideal negative solution was calculated respectively, and the close-degree between each mission and the ideal solution was obtained. The close-degree set is {*C*_26_*,C*_27_*,C*_28_*,C*_29_*,C*_30_}, and the corresponding values are {0.376, 0.613, 0.217, 0.510, 0.466}. Through the steps above, the quantification values of each mission priority were calculated. The mission priority values from mission T26 to mission T30 are 3, 6, 2, 5 and 4, respectively. The higher the value is, the higher the priority of the mission. The impact factors of 25 initial missions were calculated via the same method, and the calculation process is not listed due to space limitations.

### 3.3. Satellite Resource Allocation

During the simulation period (from 2018/08/24 00:00 to 2018/08/24 14:00), the satellite parameters were imported from STK, and the visible time window can be obtained by visibility analysis (Table 10). For the initial scheduling plan, the priority of each initial mission was calculated. Then, to maximize the objective function in Section 2.3.2, the hybrid genetic tabu search algorithm was used to solve the problem. By comparing several experimental results, we choose the following settings for this scenario. The population size of genetic algorithm was set to 50, the crossover probability was 0.6, the mutation probability was 0.1, the maximum number of iterations of genetic algorithm was 300; the length of tabu table was 10, the neighborhood size was 10, and the maximum number of iterations of tabu search was 200. Then, the initial scheduling plan was obtained, as shown in Table 11.

Before releasing the initial scheduling plan, five newly arrived emergency missions to be allocated were added before the missions were executed. After the priority calculation, the insertion, reallocation, replacement and deletion for newly arrived missions were performed. The scheduling plan of newly arrived missions was then obtained, as shown in Table 12.

In the initial scheduling plan, missions T4 and T17 were not allocated satellite resources due to the limitation of the time window, objective function maximization or some other constraints. After the new missions were added, the scheduling plan shows that mission T27 was directly inserted into the initial scheduling plan of satellite S3, mission T26 was inserted into the initial scheduling plan of satellite S3 through the reallocation operation, the affected mission T10 was scheduled again by satellite S3, and the scheduling time was from 12:18:23 to 12:19:38. Missions T29 and T30 were respectively scheduled by satellites S2 and S1 through the replacement operation, and the affected missions T21 and T15 were deleted. Mission T28 has a small mission priority and was not allocated satellite resources under the condition that the global objective was maximized.

**Figure 3:**
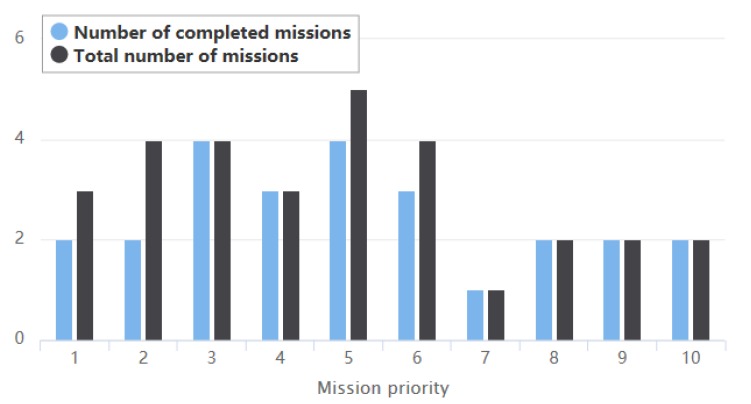
The completed number and total number of missions with different priorities in the dynamic scheduling plan.

**Figure 4:**
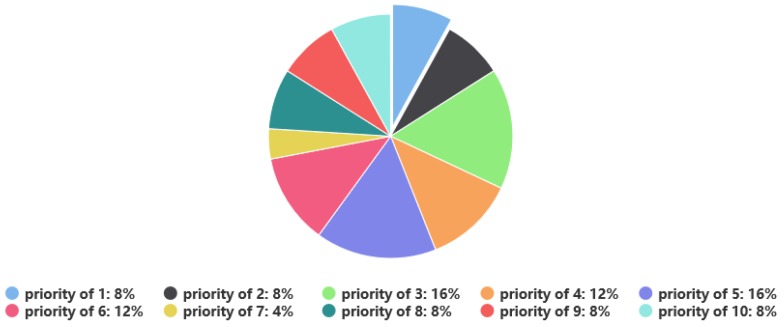
The ratio of missions with different priorities to all completed missions in the dynamic scheduling plan.

Figure 3 illustrates the statistical results of completed missions under the scenario with 30 missions. It can be seen that a total of 25 missions were completed after the dynamic scheduling due to the arrival of new missions. The missions with high priority (priorities level of 7, 8, 9 and 10) were all executed. Some missions with low priority (priorities level of 1, 2, 5 and 6) were not executed, because these missions were not allocated satellite resources during the initial scheduling or were replaced or deleted by a mission with higher priority in the dynamic scheduling. Overall, the proposed method guarantees a high execution rate for high-priority missions. The scheme change rate is 12% by calculating the model performance evaluation, so it is very helpful to guarantee the stability of the initial scheduling plan. Figure 4 shows that among the 25 executed missions, the missions with priorities higher than 6 account for 28% of the total number of completed missions, achieving a satisfactory scheduling effect.

### 3.4. Evaluation of the Scheduling Plan Result

**Figure 5:**
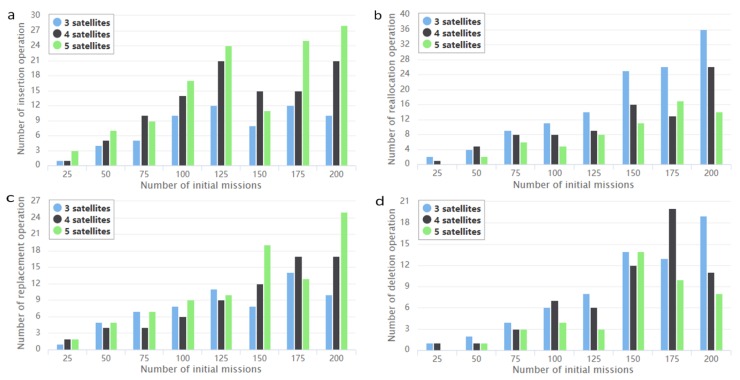
The numbers of the four types of operations performed in dynamic scheduling under different simulation scenarios. (**a**–**d**) are the scheduling results for insertion, reallocation, replacement and deletion operations.

In this section, we have conducted several numerical experiments to verify the effectiveness of the proposed method. For the model, 24 scenarios were setup and the number of initial missions contained in these scenarios changed from 25 to 200, with an increase step of 25. The corresponding number of newly arrived missions changed from 5 to 75, with an increase step of 10. Simulation experiments were conducted in scenarios with 3, 4 and 5 satellite resources. The frequency of the insertion, reallocation, replacement and deletion operations for emergency missions in different scenarios was counted to obtain Figure 5. 

It shows that for the same number of missions, as the number of satellite resources increases, the number of insertion operation notably increases, and the number of deleted missions decreases, which reduces the adjustment to the initial scheduling plan. The number of reallocation operation performed for missions is generally higher than that of replacement operation, which reduces the probability of deleting the initial missions. 

The model evaluation function defined in Section 2.5 was performed to evaluate the scheduling results for 24 different scenarios, and the computational complexity was measured using elapsed CPU time (Table 13). The evaluation indicators of the scheduling plans indicate that for the same number of satellite resources, as the number of missions increases, the mission completion rate and priority execution rate exhibit decreasing trends. 

When the number of missions is less than 100, the mission completion rate and the priority execution rate are both greater than 0.8 in different scenarios, achieving a better scheduling effect. In the scenario with 25 initial missions, five newly arrived missions and five satellites, the maximum value of the evaluation function is 11.40. Because the numbers of insertion and reallocation operations in this scenario are 3 and 2, respectively, the scheme change rate is small. However, when the number of missions is more than 100, the results for each evaluation indicator are poor. We can see that the running time of initial scheduling generally increases with the number of missions, with a maximum of 564.72 s. In contrast to the initial scheduling, the computation time for the dynamic scheduling are quite short, because the satellite resources are allocated based on initial scheduling and mission priority. However, the running time increases significantly with mission size because more different operations are needed in the iteration. It is worth mentioning that the mission priority execution rate is always larger than the mission completion rate, this phenomenon illustrates that the proposed method increases the execution probability of high-priority missions. The above results show that the proposed model is practicable and can meet the need of real-time processing with low time cost, especially for solving small and medium-scale problems. However, due to the mission priority set in this paper, the resource allocation strategy may be unfair, so some processes may wait for a long time. From the mission completion rate in Table 13, some missions were delayed the execution process because of their low priority or low revenue, which may lead to the problem of starvation. This situation could be solved by increasing the number of satellite resources, so as to increase the chance of each mission being executed. In addition, it can adjust the weight allocation of the optimization objective function, so that as many missions as possible can be executed before deadline.

## 4. Conclusions and Future Work

Dynamic emergency scheduling of Earth observation satellites is of great significance for the efficient use of satellite resources to obtain ground image data under emergency conditions. This paper studies the satellite imaging scheduling problem under emergency conditions and proposes a set of reasonable mission priority calculation method based on seven impact factors. Considering the constraints in the scheduling process, a multi-objective mathematical programming model of multi-satellite dynamic emergency scheduling based on mission priority is established. This model can be used to solve the scheduling problem of newly arrived emergency missions. In the simulation experiment, complex scenarios with different mission types and different satellites were set, and the priority levels of different missions were obtained with a calculation model of mission priority. It was also used as the heuristic information to solve the constrained scheduling problem. In the multi-objective optimization, the hybrid genetic tabu search algorithm used in this paper effectively overcomes the disadvantages of the weak local search ability of the genetic algorithm and strong dependence on the initial solution of tabu search. Therefore, this hybrid algorithm avoids a large number of useless iterations when approaching the best solution in the late stage, thereby improving the efficiency of the algorithm. In the simulation scenarios with different numbers of initial missions and newly arrived missions, the analysis results show that the probability of executing high-priority missions increases because the model considers the calculation of mission priority. Thus, a reasonable initial scheduling plan for mission request can be established under emergency conditions, and the effect of satellite resource scheduling can be improved. In case of newly arrived emergency missions, a feasible solution with low time cost can be given by using dynamic scheduling algorithm based on the mission priority. Overall, the proposed model yields high scheduling revenues and low scheduling costs, maintains the stability of the initial scheduling plan under the condition of fast scheduling, so it is suitable for solving multi-satellite dynamic mission scheduling problems.

In practical applications, the constraints of satellite mission scheduling are very complex. In some cases, manual intervention is needed for obtaining the current satellite scheduling plan, such as the collection of mission request lists, the selection of satellite resources, and the weight setting of optimization objective function. In the emergency situation, the operators may need to adjust the scheme according to the latest planning configuration before it releases, including deleting observation missions and adding new missions. However, finding the balance between manual intervention and automation is a perennial challenge. In future work, if some manual intervention can be transformed into decision-making conditions of the scheduling system through learning mechanism, it will be more capable to meet the demand. Furthermore, we will analyze more complex scheduling problems on this basis and further refine the model. Additionally, more constraints will be considered to make the scheduling problems more comprehensive.

## Figures and Tables

**Table 1:** Impact factors of mission priority.

ID	Impact Factor	Data Type	Formula	Explanation
F1	Imaging mission level	Real Number	$e_{i}=1/l_{i}$	$e_{i}$ is the rating value of emergency mission *i*, and $l_{i}$ is the level of the emergency mission.
F2	Type of observation image	Enumerated type	—	—
F3	Visibility of target to satellite	Real Number	$d_{i}=1/n_{i}$	*d_i_* is the visibility rate of mission *i* to satellite in its observation time window, and *n_i_* is the number of available satellites in the observation time window.
F4	Execution urgency degree	Real Number	$u_{i}=\left(t_{r}^{i}-t_{b}^{i}\right)/\left(t_{e}^{i}-t_{b}^{i}\right)$	$u_{i}$ is the execution urgency degree of mission *i*, $t_{b}^{i}$ is the start time of mission *i*, $t_{e}^{i}$ is the end time of mission *i*,$t_{r}^{i}$ is the remaining execution time of mission *i*.
F5	Type of mission	Enumerated type	—	—
F6	Mission conflict degree	Real Number	*C_i_ = n_c_*	*C_i_* is the conflict degree of mission *i*, and *n_c_* is the number of missions competing with the same satellite resource as mission *i*.
F7	Revenue of imaging mission	Real Number	$p_{i}=p_{i}^{b}/cc_{i}$ $p_{i}^{b}=k\times e_{i}$	*p_i_* is the imaging revenue of mission *i*,$p_{i}^{b}$ is the basic revenue, *cc_i_* is the parameter of cloud cover obtained by the International Satellite Cloud Climatology Project (ISCCP) [28], and *k* is an adjustment coefficient.

**Table 2:** Parameter and label definitions.

Notation	Definition	Notation	Definition
*S*	*S* = {*s*_1_,*s*_2_,…,*s_n_*}, where *S* is the set of satellites and *n* represents the number of satellites.	*N_ij_*	*N_ij_* represents the number of visibility time windows of the sensor of satellite *s_j_* to mission *t_i_*.
*T*	*T* = {*t*_1_,*t*_2_,…,*t_m_*}, where *T* is the set of missions and *m* represents the number of missions. [*tsb_i_,tse_i_*] represents the validity time window of mission *t_i_*.	*p_i_*	*p_i_* is the priority level of mission *t_i_*.
*GSW_j_*	*GSW_j_* is the set of the ground station time windows, representing the time window set of satellite *s_j_* for all ground stations.	*w*(*t_i_*)	*w*(*t_i_*) is the imaging revenue of mission *t_i_*.
$SA_{ij}^{l}$	$SA_{ij}^{l}$ is the angle of the side swing, representing the side swing angle of the sensor of satellite *s_j_* to the *l*-th visible time window of mission *t_i_*.	*ST_ij_*	*ST_ij_* = [*bst_ij_,est_ij_*], where *ST_ij_* represents the execution period after mission scheduling, *bst_ij_* is the scheduled start time and *est_ij_* is the scheduled end time of mission *t_i_* for satellite *s_j_*.
$OS_{ij}^{l}$	$OS_{ij}^{l}=\left[bos_{ij}^{l},eos_{ij}^{l}\right]$ where $OS_{ij}^{l}$ represents the *l*-th time window of the sensor of satellite *s_j_* to mission *t_i_*, $bos_{ij}^{l}$ is the start time of time window, and $eos_{ij}^{l}$ is the end time of time window.	*p_ijl_*(*tm*)	*p_ijl_*(*tm*) represents the imaging duration. At time *tm*, mission *t_i_* is executed by satellite *s_j_* in the *l*-th visible time window.

**Table 3:** Function definitions in the constraints.

Function	Definition
*CapLen*(*t_i_*)	The shooting duration, which is related to the mission requirements and is obtained before scheduling.
*IS*(*OS_ij_*)	The start-up time of satellite *s_j_*, which can be calculated from the specific execution time after scheduling.
*CS*(*OS_ij_*)	The shutdown time of satellite *s_j_*, which can be calculated from the specific execution time after scheduling.
*SunAngle*(*t_,_*,*tm*)	The solar elevation angle, which can be calculated from the geographical position of mission *t*_i_ and the time *tm*.
*ObserAngle*(*t_i_*,*s_j_*,*tm*)	The observation angle, which can be calculated from the geographical position of mission *t_i_*, the path of satellite *s_j_* and the time *tm*.
*StorCap*(*t_i_*,*v_j_*)	The storage capacity of satellite *s_j_*, which is occupied by the observation mission *t_i_* arranged between any two data transmission windows *w*_1_ and *w*_2_. It can be calculated from the planned observation mission *ti* and the speed of reading and writing *v_j_* of satellite *s_j_*.
*NUM*(*E*)	The number of on-off switches for satellite *s_j_*, which can be calculated from the start-up time and shutdown time of satellite *s_j_*.
*WP*(*MT_l_*)	The working wave of satellite s*_j_* which is related to mission *t_i_*.
*urgent*(*t_i_*)	An indicator represents whether mission *t_i_* is an urgent mission.

**Table 4:** Processing chain for the hybrid genetic tabu search algorithm.

Hybrid Genetic Tabu Search Algorithm	
Input	(1) the maximum iteration number of genetic algorithm *i_ga–max_*; (2) the maximum iteration number of tabu search *i_ts–max_*; (3) the population size *n*; (4) the crossover probability *p_c_*; (5) the mutation probability *p_m_*; (6) the length of tabu table *l*; (7) the neighborhood size *n*; (8) the neighborhood function *F(x*).
Processing chain	(1) Determine the chromosome coding method. Chromosome coding is a series of segments, each of which represents the mission scheduling for a satellite payload type. In each segment, the execution window of each mission is arranged by number to form the chromosome coding of this segment. The chromosome coding template is defined as $u_{i}=\left\{t_{i},w_{i}^{q},st_{i}\right\}$, where $w_{i}^{q}$ is the visible window of mission *t_i_*, *st_i_* is the start time of execution, and the coding template is represented as a vector ν, where ν = {*u*_1_,*u*_2_,…,*u_n_*}.
	(2) Initialize the population. An initial population of size *N* is randomly generated. The initial generation *t* is set to 0, *m* chromosomes are randomly generated as $x_{t}^{1},x_{t}^{2},\ldots ,x_{t}^{m}$, and the optimal value is set as *X_best_*.
	(3) Calculate the value of the fitness function. The objective function is taken as the fitness function of chromosome, and the fitness function value $f(x_{t}^{1}),f\left(x_{t}^{2}\right),\ldots ,f\left(x_{t}^{m}\right)$ of each chromosome is calculated in the population. If *f*_(_*X_best_*) < $f(x_{t}^{i})$, *i* = 1,2,…,*m,* then *X_best_* = $x_{t}^{i}$.
	(4) If *t* < *i_max_*, then *t* = *t* +1, otherwise, the iteration ends.
	(5) Perform selection operation on the population. The roulette wheel selection method is used to select *m* chromosomes from the population as the parent of the next generation, and the probability of each chromosome being selected is $P_{i}=f\left(x_{t}^{i}\right)/\overset{m}{\underset{j=1}{\sum }}f\left(x_{t}^{j}\right)$, *i* = 1,2,…,*m*. In the selection process, the best member of the previous generation is retained in the next generation.
	(6) Perform crossover operation on the population. Chromosomes are crossed by single point crossover according to crossover probability *P_c_*.
	(7) Perform mutation operation for the population. According to the mutation probability *P_m_*, individuals in the population are mutated to produce a new generation.
	(8) Perform a tabu search for the new generation and determine whether the termination criterion is satisfied. If the criterion is satisfied, the best solution is output; otherwise, the candidate solution is determined by the neighborhood function *F*(*x*), which generates the neighborhood of the current solution.
	(9) Assess the merit of the candidate solution based on the fitness function and whether it satisfies the aspiration criterion. If the criterion is satisfied, it is assigned to the current solution and the best solution found-so-far, the tabu length is set again, and the tabu length of other elements is updated. Otherwise, the sub-best solution is taken as the current solution and added to the tabu table to replace the object that first entered the tabu table. The lengths of other elements in the tabu table are updated.
Output	When the maximum iteration number *i_ts–max_* is reached, the best solution is output.

**Table 5:** Initial mission parameters.

Mission ID	Observation Duration (sec)	Geographical Position	Imaging Mission Level	Observation Image Type	Type of Mission
T1	110	(90° E, 30° N)	NII	infrared	land static target
T2	90	(30° W, 20° S)	SI	visible light	maritime static target
T3	200	(110° E, 45° N)	HIII	visible light	land static target
T4	150	(90° W, 60° N)	SII	visible light	land moving target
T5	210	(1° W, 5° S)	AIV	infrared	maritime static target
T6	60	(105° W, 25° N)	HII	visible light	land static target
T7	90	(150° E, 55° S)	NI	infrared	maritime static target
T8	130	(138° W, 15° N)	AIII	microwave	maritime moving target
T9	150	(55° E, 63° N)	SIV	visible light	land moving target
T10	170	(14° W, 70° N)	NII	visible light	maritime moving target
T11	170	(41° E, 32° N)	HIV	visible light	land moving target
T12	220	(63° W, 28° S)	AIII	infrared	land static target
T13	100	(170° W, 54° S)	AIV	infrared	maritime static target
T14	160	(73° E, 26° S)	SI	visible light	maritime moving target
T15	130	(42° E, 59° N)	NII	visible light	land static target
T16	200	(130° E, 21° S)	HII	visible light	land static target
T17	140	(20° E, 25° N)	NIV	microwave	land moving target
T18	80	(46° W, 38° N)	SI	infrared	maritime moving target
T19	120	(49° W, 11° S)	AII	visible light	land static target
T20	200	(110° E, 68° N)	HIV	infrared	land static target
T21	60	(118° W, 45° N)	SIII	visible light	land moving target
T22	130	(140° W, 64° N)	SI	infrared	land static target
T23	70	(14° E, 52° S)	AII	microwave	maritime moving target
T24	110	(114° W, 15° S)	NII	microwave	maritime moving target
T25	190	(36° E, 15° N)	HIII	visible light	land static target

Notes: N refers to a natural disaster, A refers to an accident disaster, H refers to a public health incident and S refers to a social security incident. I~IV represent the disaster level.

**Table 6:** Newly arrived mission parameters.

Mission ID	Observation Duration (sec)	Geographical Position	Imaging Mission Level	Observation Image Type	Type of Mission
T26	80	(65° E, 4° N)	SII	infrared	maritime static target
T27	170	(69° W, 4° N)	HI	visible light	land static target
T28	110	(64° E, 51° N)	NIV	microwave	land static target
T29	150	(15° W, 14° N)	SI	visible light	land moving target
T30	100	(73° E, 12° S)	AIII	visible light	maritime moving target

**Table 7:** Orbit parameters of satellite.

Orbit Parameters	Satellite		
	S1	S2	S3
Eccentricity *e*	0.2030	0.24008	0.1353
Inclination *i* (°)	97.9072	97.7823	98.1956
Right ascension of the ascending node *Ω* (°)	324.7362	321.0446	322.0663
True anomaly *ω* (°)	49.6914	0.8425	69.8332
Mean anomaly *M* (°)	310.4478	359.2829	290.3028
Number of circles per day	14.84971693	14.79898507	14.58551423

**Table 8:** Information of priority impact factors for newly arrived missions.

Mission ID	F1	F2	F3	F4	F5	F6	F7
T26	0.50	infrared	0.50	0.228	land static target	1	485
T27	1.00	visible light	1.00	1	maritime moving target	0	1250
T28	0.25	microwave	0.50	0.698	land static target	1	357
T29	1.00	visible light	0.33	0.417	land moving target	2	714
T30	0.33	visible light	0.50	0.742	maritime moving target	1	1100

**Table 9:** Standard matrix of priority impact factors for newly arrived missions.

Mission ID	F1	F2	F3	F4	F5	F6	F7
T26	0.333	0.000	0.254	1.000	0.000	0.500	0.143
T27	1.000	1.000	1.000	0.000	1.000	0.000	1.000
T28	0.000	0.254	0.254	0.128	0.000	0.500	0.000
T29	1.000	1.000	0.000	0.413	0.107	1	0.400
T30	0.107	1.000	0.254	0.103	1.000	0.500	0.832

**Table 10:** Visible time window for the initial missions and new missions.

Mission ID	Mission Priority	S1		S2		S3	
		Start Time	End Time	Start Time	End Time	Start Time	End Time
T1	6	06:13:44	06:14:27	09:37:15	09:38:51	13:47:54	13:48:36
T2	9	02:34:47 12:15:08	02:37:01 12:17:42	04:15:30 10:03:41	04:17:28 10:04:58	04:53:17 13:03:48	04:55:46 13:05:21
T3	8	13:58:00	14:01:12	07:43:11 13:21:43	07:44:36 13:23:06	07:29:04	07:32:17
T4	6	09:34:15	09:36:03	11:21:17	11:24:01	09:18:34	09:19:56
T5	3	08:23:09	08:24:38	13:25:09	13:26:53	02:06:25 10:25:09	02:09:56 10:26:59
T6	4	01:46:01 11:27:31	01:48:33 11:28:53	10:39:16	10:42:23	05:56:17	05:58:09
T7	5	13:40:15	13:42:36	06:12:08 11:44:12	06:15:19 11:45:53	13:37:45	13:38:39
T8	1	11:08:45	11:10:37	00:59:27 06:41:08 12:07:57	01:02:16 06:44:12 12:09:25	10:29:12	10:32:47
T9	5	05:22:53	05:24:02	13:42:06	13:43:52	06:48:17	06:50:44
T10	3	08:59:23	09:01:24	02:57:23	02:59:11	12:18:07	12:19:43
T11	2	01:35:14 11:07:34	01:36:45 11:09:05	03:15:43 09:02:40	03:17:04 09:04:28	00:48:26 09:03:46	00:52:04 09:06:37
T12	4	12:02:56	12:04:27	00:25:37 06:14:07	00:26:42 06:17:13	07:05:36	07:08:20
T13	5	08:24:09	08:26:42	09:03:46	09:06:12	12:37:09	12:38:25
T14	10	00:11:34 09:27:56	00:13:52 09:30:41	10:08:57	10:11:51	01:41:58 09:58:45	01:45:04 10:02:37
T15	2	13:41:20	13:42:47	09:18:02	09:21:07	10:15:36	10:17:52
T16	7	07:52:21	07:53:48	12:30:41	12:33:26	06:38:45	06:41:48
T17	1	09:18:03	09:20:25	09:13:37	09:14:09	07:37:42	07:39:23
T18	8	05:38:42	05:39:57	03:24:15 08:58:29	03:25:58 09:01:12	05:03:32 12:46:34	05:06:25 12:48:02
T19	10	00:15:42 10:12:04	00:17:06 10:13:47	07:41:23 12:13:26	07:42:16 12:15:03	09:28:07	09:31:45
T20	1	04:49:13	04:51:04	10:38:46	10:41:05	10:46:44	10:47:36
T21	5	10:27:56	10:28:47	07:26:13	07:29:38	09:03:44	09:05:47
T22	3	07:03:56	07:05:14	12:38:26	12:39:47	13:05:57	13:09:35
T23	6	10:51:46	10:52:34	10:26:53	10:29:41	11:57:03	11:59:35
T24	9	12:06:34	12:09:25	11:27:14	11:29:21	08:33:41	08:36:23
T25	2	13:35:26	13:36:12	04:53:19 10:24:09	04:55:40 10:27:22	10:16:18	10:19:00
T26	3	09:46:04	09:48:57	01:33:49 08:04:19	01:34:52 08:05:57	09:35:43	09:37:22
T27	6	06:04:19	06:07:28	12:41:04	12:43:32	00:49:25 09:01:30	00:50:58 09:04:13
T28	2	11:58:01	11:59:33	05:20:41 11:04:18	05:21:59 11:05:48	13:08:01	13:11:28
T29	5	02:28:18 12:05:52	02:30:57 12:07:39	07:27:34	07:28:52	04:26:19 12:16:31	04:28:06 12:17:50
T30	4	13:41:03	13:43:18	04:03:55 09:35:21	04:05:24 09:36:49	12:39:47	12:41:40

**Table 11:** Initial scheduling plan.

Mission ID	Satellite	Start Time	End Time
T1	S3	13:47:54	13:48:17
T2	S2	04:15:30	04:17:09
T3	S2	13:21:54	13:22:58
T5	S1	08:23:09	08:24:30
T6	S3	05:56:35	05:58:01
T7	S2	06:12:08	06:15:14
T8	S2	12:07:57	12:09:14
T9	S1	05:22:59	05:23:47
T10	S3	12:18:23	12:19:38
T11	S1	01:35:14	01:36:40
T12	S3	07:05:36	07:08:13
T13	S2	09:03:54	09:05:53
T14	S2	10:08:57	10:11:51
T15	S1	13:41:37	13:42:34
T16	S3	06:38:45	06:41:48
T18	S2	03:24:29	03:25:40
T19	S3	09:28:23	09:31:33
T20	S1	04:49:34	04:51:04
T21	S2	07:26:13	07:29:05
T22	S1	07:04:12	07:05:14
T23	S3	11:57:16	11:59:27
T24	S1	12:06:45	12:09:13
T25	S3	10:16:18	10:18:27

**Table 12:** Scheduling plan of newly arrived missions.

Mission ID	Satellite	Scheduling Operation	Influenced Mission	Start Time	End Time
T26	S3	Reallocation	T10	09:35:56	09:37:22
T27	S3	Insertion	None	00:49:25	00:50:58
T28	-	Deletion	None	-	-
T29	S2	Replacement	T21	09:03:54	09:04:38
T30	S1	Replacement	T15	13:41:03	13:43:05

**Table 13:** Evaluation of scheduling results under different mission sizes.

Number of Initial Missions	Number of Newly Arrived Missions	Number of Satellites	Mission Completion Rate	Mission Priority Execution Rate	Scheme Change Rate	Running Time(s) of Initial Scheduling	Running Time(s) of Dynamic Scheduling	Evaluation Function
25	5	3	0.83	0.89	0.12	10.43	0.32	6.16
		4	0.88	0.92	0.12	10.97	0.38	6.75
		5	0.94	0.97	0.08	11.25	0.42	11.40
50	15	3	0.82	0.85	0.18	37.68	1.21	3.87
		4	0.85	0.88	0.18	40.43	1.53	4.16
		5	0.92	0.94	0.14	45.97	1.88	6.18
75	25	3	0.82	0.86	0.21	62.06	5.87	3.36
		4	0.87	0.91	0.16	78.32	7.00	4.95
		5	0.90	0.93	0.17	86.27	7.98	4.92
100	35	3	0.81	0.84	0.19	144.14	12.07	3.58
		4	0.83	0.85	0.16	157.93	15.05	4.41
		5	0.87	0.89	0.14	173.75	17.62	5.53
125	45	3	0.79	0.82	0.20	162.67	26.87	3.24
		4	0.76	0.78	0.12	210.40	35.11	4.94
		5	0.80	0.82	0.14	238.45	42.65	4.69
150	55	3	0.73	0.70	0.22	229.70	35.16	2.32
		4	0.78	0.76	0.19	272.59	43.78	3.12
		5	0.81	0.80	0.20	326.01	54.59	3.24
175	65	3	0.64	0.72	0.23	294.24	79.13	2.00
		4	0.62	0.69	0.19	360.35	93.71	2.25
		5	0.75	0.73	0.17	429.31	122.84	3.22
200	75	3	0.61	0.71	0.23	371.35	135.37	1.88
		4	0.65	0.74	0.21	485.66	160.82	2.29
		5	0.72	0.78	0.20	564.72	185.03	2.81
